# Temporal Responses of Microbial Communities to Anaerobic Soil Disinfestation

**DOI:** 10.1007/s00248-019-01477-6

**Published:** 2019-12-23

**Authors:** Amisha T. Poret-Peterson, Nada Sayed, Nathaniel Glyzewski, Holly Forbes, Enid T. González-Orta, Daniel A. Kluepfel

**Affiliations:** 1 USDA-ARS Crops Pathology and Genetics Research Unit, University of California, Davis, USA; 2 University of California Davis Medical Center, Sacramento, CA USA; 3 Green Leaf Lab, Sacramento, CA USA; 4 Department of Biological Sciences, California State University, Sacramento, CA USA

**Keywords:** Anaerobic soil disinfestation, Soil microbiome, Plant disease, Organic amendment, Management tool

## Abstract

Anaerobic soil disinfestation (ASD) is an organic amendment-based management tool for controlling soil-borne plant diseases and is increasingly used in a variety of crops. ASD results in a marked decrease in soil redox potential and other physicochemical changes, and a turnover in the composition of the soil microbiome. Mechanisms of ASD-mediated pathogen control are not fully understood, but appear to depend on the carbon source used to initiate the process and involve a combination of biological (i.e., release of volatile organic compounds) and abiotic (i.e., lowered pH, release of metal ions) factors. In this study, we examined how the soil microbiome changes over time in response to ASD initiated with rice bran, tomato pomace, or red grape pomace as amendments using growth chamber mesocosms that replicate ASD-induced field soil redox conditions. Within 2 days, the soil microbiome rapidly shifted from a diverse assemblage of taxa to being dominated by members of the Firmicutes for all ASD treatments, whereas control mesocosms maintained diverse and more evenly distributed communities. Rice bran and tomato pomace amendments resulted in microbial communities with similar compositions and trajectories that were different from red grape pomace communities. Quantitative PCR showed nitrogenase gene abundances were higher in ASD communities and tended to increase over time, suggesting the potential for altering soil nitrogen availability. These results highlight the need for temporal and functional studies to understand how pathogen suppressive microbial communities assemble and function in ASD-treated soils.

## Introduction

Anaerobic soil disinfestation (ASD) is an organic amendment-based pre-plant treatment for the control of plant pathogens in a variety of cropping systems [1–4]. It is a sustainable alternative to chemical fumigation of soil using compounds such as 1,3-dichloropropene and chloropicrin for the management of soil-borne plant diseases [5]. ASD involves the generation of anaerobic conditions in soil through addition of a readily available carbon source (i.e., agricultural materials), irrigation of soil to field capacity, and sealing of soil with a gas impermeable tarp [1, 4]. Ultimately, ASD induces changes in soil physicochemistry and the microbiome that result in the reduced viability of many plant pathogens [5–8].

ASD using different carbon substrates has been shown to be effective at controlling plant parasitic nematodes [9] and several microbial plant pathogens: *Agrobacterium tumefaciens*, *Fusarium oxysporum*, *Ralstonia solanacearum*, *Rhizoctonia solani*, *Pythium* spp., and *Verticillium dahliae* [5, 6, 10–13]. Carbon substrates used to initiate ASD include agricultural by-products (e.g., ethanol, molasses, rice bran, seed meals, and wheat bran), cruciferous cover crops, and composted poultry litter [2, 3, 14]. Other agricultural by-products are also being examined for their effectiveness as ASD substrates, including tomato pomace, red grape pomace, nuts and shells from almond, walnut, and pistachio in order to reduce costs of the process and increase its adoption as a management tool [15]. Despite the carbon source used, ASD-mediated pathogen control has been found to involve the products of microbial fermentation (i.e., volatile fatty acids, acetate, butyrate, etc.), lowered soil pH and redox potential, the release of Mn^2+^ and Fe^2+^, and elevated soil temperature [6, 8, 10, 16–18].

The resulting soil microbiome appears to be a critical factor in how ASD mediates pathogen suppression, as anaerobic conditions alone may not be sufficient to cause reduction in the survival of plant pathogens [1]. In ASD-treated soils, bacterial taxa associated with Acidobacteria, Bacteroidetes, and Firmicutes become predominant and their physiological activities (i.e., fermentation) contribute substantially to the suppressiveness or lethality of ASD for plant pathogens [6–8]. ASD is also a disturbance to the soil ecosystem and offers opportunities to examine the response of soil microbes to the imposition of anaerobic conditions, input of organic carbon, and the re-assembly of the soil microbiome [19]. Previously, we reported that ASD implemented with different agricultural by-products did not yield communities that were strongly structured as a function of carbon substrate after 4 and 5 weeks of ASD treatment [15]. Instead, ASD resulted in communities consisting of a core group of taxa belonging to the Firmicutes classes Clostridiales and Selenomonadales. We also showed the most abundant taxa in ASD-treated soils have the genomic potential to produce compounds known to inhibit the growth of plant pathogens and perform biological nitrogen fixation. In this study, we track microbial community changes over time (3 weeks) in response to ASD initiated with rice bran, tomato pomace, or red grape pomace using soil mesocosms under controlled conditions. We also included control soil mesocosms that were anaerobic, but did not receive carbon input. Our specific aims were to (1) test soil mesocosms for their ability to allow the generation and maintenance of soil redox potentials obtained in ASD field trials and (2) determine if the structure and composition of the soil microbiome is strongly shaped by the choice of carbon substrate at early time points during ASD treatment.

## Material and Methods

### Soil Mesocosm Design, Setup, and Sampling

We conducted an ASD trial using soil mesocosms under controlled conditions. Treatments consisted of four replicates of a no carbon control (NCC) and ASD using rice bran (RB), red grape pomace (RGP), or tomato pomace (TP) as the carbon sources. Soil mesocosms were incubated in an unlit, ventilated, and temperature-controlled (27 to 29 °C) growth chamber at the University of California, Davis, CA, USA from 23 August to 13 September 2017. Soil used in this study was collected from the University of California, Kearney Agricultural Research Center located in Parlier, CA, USA. The soil at the location is Hanford series sandy loam with near-neutral pH (7.3), total organic matter content of ~ 1%, and extractable phosphate, nitrate, and ammonium in the amounts of 0.3, 5.7, and 1.7 mg L^−1^, respectively (Albu et al., unpublished). A small portion (~ 1 g) of this soil was frozen on dry ice and ethanol for characterization of the pre-treatment community.

We constructed the soil mesocosms from polyvinyl chloride (PVC) tubes that were 15.2 cm in diameter and 30.5 cm in length. To facilitate repeated sampling, three 1.5-cm holes were drilled at equal distances around the tube perimeter at a height of 10.2 cm from the bottom of each tube. The holes were plugged with 20-mm butyl rubber stoppers (Bellco Glass, Inc., Vineland, NJ, USA) and the PVC tube bottoms were covered using nylon mesh (20-μm pore size). Soil was adjusted to 20% moisture content immediately before dispensing into the PVC mesocosms. Each mesocosm was packed with soil to a height of 25.4 cm. For ASD treatments (RB, RGP, or TP), the upper 15.2 cm of soil was removed and placed into concrete mixing bins. The carbon source was homogenously mixed into the soil at an application rate of 20.2 t ha^−1^ and the soil was then placed back into the mesocosms. The upper 15.2 cm of soil from NCC mesocosms were treated as described above except no carbon source was added during the mixing process. Oxidation reduction potential (ORP) sensors (Model S500CD-ORP, Sensorex, Garden Grove, CA, USA) attached to CR1000 data loggers (Campbell Scientific, Logan, UT, USA) programmed for hourly measurements were placed into each mesocosm to a depth of 15.2 cm. Approximately 200 mL of distilled water was then poured on top of the soil column and all mesocosms were sealed with a transparent totally impermeable film (TIF, VAPORSAFE, Raven Engineered Films, Sioux Falls, SD, USA). Each mesocosm was placed in separate plastic bins (25.4-cm diameter and 12.7-cm height) that were filled with distilled water to a height of 10.2 cm; the water level in the plastic bins was maintained via periodic additions of distilled water. It took approximately 1.5 h to set up the mesocosms from packing to activation of the ORP sensors. Mesocosms were arranged in a randomized block design.

The control and ASD-treated soils were incubated for a total of 21 days and sampled at three time points: 2, 9, and 21 days post ASD initiation. Soil cores were collected through a different lateral sampling port at each time point using a modified 5-mL syringe. The soil cores were immediately frozen on a dry ice and ethanol slurry and stored at − 80 °C until nucleic acid extraction.

To estimate soil redox potential (Eh) in the mesocosms, ORP sensor readings were converted to standard hydrogen electrode output by adding 200 mV. Cumulative anaerobicity or the total time that soil Eh is under + 200 mV during ASD was calculated by summing the difference between soil Eh and the critical reduction potential (+ 200 mV) over the 3-week trial. Analysis of variance (ANOVA) was used to test for significant differences in cumulative anaerobicity between treatments using R version 3.5.0 [20].

### Nucleic Acid Extraction, 16S rRNA Gene Sequencing, Bioinformatic Processing, and Statistical Analyses

DNA was purified from ~ 0.5-g soil samples using the PowerLyzer PowerSoil DNA Isolation Kit (Mo Bio Laboratories, Inc., Carlsbad, CA, USA) following the manufacturer’s protocol. DNA integrity and purity was confirmed via gel electrophoresis, absorbance, and PCR amplification. The quantity of DNA was measured via Qubit™ dsDNA High Sensitivity assay (Invitrogen, Carlsbad, CA, USA).

RNA was extracted from day 9 samples using the RNeasy® PowerSoil Total RNA Kit (QIAGEN, Inc., Germantown, MD, USA) following the manufacturer’s protocol with the following exceptions. The contents of the PowerBead tubes were transferred to 15-mL bacteria lysing CK01 tubes (Bertin Instruments, Rockville, MD, USA). Soil samples (~ 2 g) and reagents were added to this tube and bead-beating performed in a Precellys Evolution homogenizer (Bertin Instruments, Rockville, MD, USA) at a speed of 6800 rpm for 45 s. Following extraction, RNA was treated with the DNase Max Kit (Mo Bio Laboratories, Inc., Carlsbad, CA, USA) and purified via the RNeasy® MinElute® Cleanup Kit (QIAGEN, Inc., Germantown, MD, USA) according to the manufacturers’ protocols. RNA integrity was checked via gel electrophoresis and RNA was quantified using the Qubit™ RNA High Sensitivity assay (Invitrogen, Carlsbad, CA, USA). Total RNA (~ 0.5 to 1 μg) was reverse-transcribed into single-stranded cDNA using random hexamers and SuperScript® IV (Invitrogen, Carlsbad, CA, USA). Single-stranded cDNA was purified using the GenElute™ PCR Clean-Up kit (Sigma-Aldrich, St. Louis, MO, USA). A portion of the total RNA was also retained for non-reverse transcribed controls, which were subjected to PCR to check for genomic DNA carryover prior to sending cDNA for sequencing; no products were obtained.

DNA extracts and cDNA were sent to the Michigan State University Research Technology and Support Facility Genomics Core for sequencing of the 16S rRNA gene (v4 region) using primer set 515F/806R [21] as described in Kozich et al [22]. We used the dada2 (version 1.4.0) pipeline implemented in R (version 3.4.1) on the USDA high-performance computing cluster Ceres to process raw sequences into amplicon sequence variants (ASVs) [23]. ASVs were taxonomically classified against the Silva v128 rRNA database [24]. Raw sequences are available in NCBI under BioProject PRJNA575041.

To test for significant differences in community structure and composition between treatments and over time, we used a repeated measures permutational analysis of variance (PERMANOVA) implemented in PRIMER version 7 with the PERMANOVA+ package [25]. The repeated measures PERMANOVA was performed on a Bray-Curtis dissimilarity matrix of normalized ASV abundances with treatment and day of sampling as fixed effects and mesocosm ID nested in treatment as a random effect. The R package vegan was used to calculate alpha diversity measures (number of ASVs, Shannon’s diversity index) for all samples and test for homogeneity of group dispersions over time for each treatment [26]. An additional measure of alpha diversity, Pielou’s evenness index, was calculated as the Shannon index divided by the natural log of ASV richness. The R package lme4 was used to test for significant differences in Shannon’s diversity and Pielou’s evenness indices using linear mixed models [27]. The R package mvabund was used to perform differential abundance tests between DNA- and cDNA-derived communities for each ASD carbon substrate [28].

### qPCR Analyses

Quantitative PCR of bacterial 16S rRNA and nitrogenase (*nifH*) genes was performed using Brilliant III Ultra-Fast SYBR® Green QPCR Master Mix (Agilent Technologies, Santa Clara, CA, USA) in a QuantStudio 6 Flex Real-Time PCR System (Applied Biosystems, Foster City, CA, USA). Bacterial 16S rRNA genes were amplified with 338F and 518R [29] and nifH genes were amplified with F2 and R6 [30] using the following thermal cycler parameters: initial denaturation at 95 °C for 3 min followed by 40 cycles of denaturation (95 °C for 20 s), primer annealing (20 s at 52 °C for *nifH* or 55 °C for 16S rRNA genes), extension (72 °C for 20 s), and fluorescence capture (80 °C for 20 s). A melt curve was collected from 60 to 95 °C following qPCR. Quantitative PCR reactions (10 μL) were run in triplicate and consisted of 1× master mix, 30 nM ROX, 0.5 μM (16S rRNA genes) or 0.8 μM (*nifH*) primer, and 400 ng μL^−1^ bovine serum albumin. For bacterial 16S rRNA genes, standard curves from 10^2^ to 10^7^ copies μL^−1^ (94 to 97% primer efficiency) were constructed from serial dilution of a PCR product of the entire 16S rRNA gene amplified from *Pseudomonas aeruginosa* PAO1. The standard curves for *nifH* ranged from 10^1^ to 10^8^ copies μL^−1^ (94% primer efficiency) and were constructed from a cloned *nifH* gene from soil. Using the R package lme4 [27], we calculated correlations between genera and *nifH* abundances using linear regression with gene copy number and treatment as fixed effects and mesocosm ID as a random factor. Correlations between genera and *nifH* abundances with a false discovery rate less than 0.05 were considered significant.

## Results and Discussion

### Development of Anaerobic Conditions in Soil Mesocosms

**Fig. 1:**
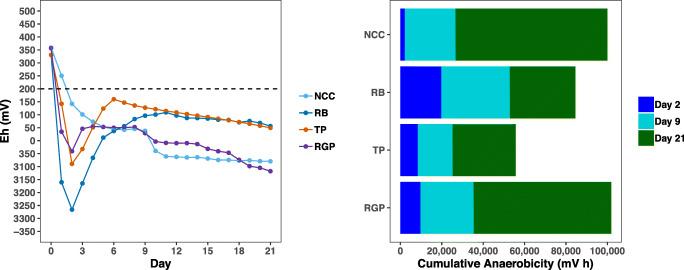
Redox potential (Eh) and cumulative anaerobicity in soil mesocosms

Soil redox potential is often used as an indicator of the effectiveness of ASD. In particular, the strength or intensity of anaerobic conditions in soil is defined as the total time Eh values remain under + 200 mV during the process (cumulative anaerobicity). This cutoff represents the shift in soil conditions from aerobic (> + 300 mV) to anaerobic, which can be further described as moderately reduced (0 to + 300 mV), reduced (0 to − 200 mV), and highly reduced (< − 200 mV) [31]. In this study, we monitored soil redox potential for 3 weeks in control (NCC) and ASD (RB, TP, RGP) mesocosms. ASD mesocosms reached reduced (RGP and TP) or highly reduced (RB) soil conditions within 2 days (Fig. 1). Soil Eh in these mesocosms then increased but remained moderately reduced or reduced throughout the incubation period. Soil Eh in the NCC mesocosms gradually declined over the course of the trial indicating reduced soil conditions. At the end of the trial, there were no significant differences in cumulative anaerobicity between NCC and ASD mesocosms (*F*_3,12_ = 2.10, *P* = 0.154).

The cumulative anaerobicity values achieved in ASD soil mesocosms in this 3-week growth chamber trial are in line with values observed in longer term (5 to 7 weeks) ASD field studies [5, 12]. This indicates the soil mesocosms used in this study sufficiently mimic in field conditions generated by ASD and are appropriate for tracking microbial community changes in response to different carbon substrates. Although we did not directly examine plant pathogen control, the cumulative anaerobicity estimates in these ASD treatments have been shown previously to effectively suppress microbial plant pathogens [12]. Cumulative anaerobicity has been used to determine thresholds for pathogen suppression (e.g., values that exceed 50,000 mV h are needed to kill *Verticillium dahliae*) [32]. Moreover, we have previously shown that *Agrobacterium tumefaciens* populations are significantly reduced within 7 days of ASD implementation with rice bran in the field where similar levels of cumulative anaerobicity were reached [12].

### Changes in Microbial Communities over Time in Soil Mesocosms

Microbial communities that develop in ASD-treated soils are shaped, to varying degrees, by the type of carbon substrate used to initiate the process [7, 15, 33, 34]. In replicated field trials of ASD stimulated with different agricultural by-products (RB, TP, molasses, or mustard seed meal), we found ASD carbon input explained a small amount of the variance in community composition (10 and 22%), but resulted in communities with much higher group dispersions than in untreated soils after 4 and 5 weeks of treatment [15]. We posited that the microbial communities would be more strongly structured by ASD carbon substrates and exhibit lower group dispersions following initiation of the process and the onset of anaerobic soil conditions. Hence, in this growth chamber study, we sequenced microbial communities at three different time points—2, 9, and 21 days—post ASD initiation with RB, TP, or RGP as carbon substrates.

**Fig. 2:**
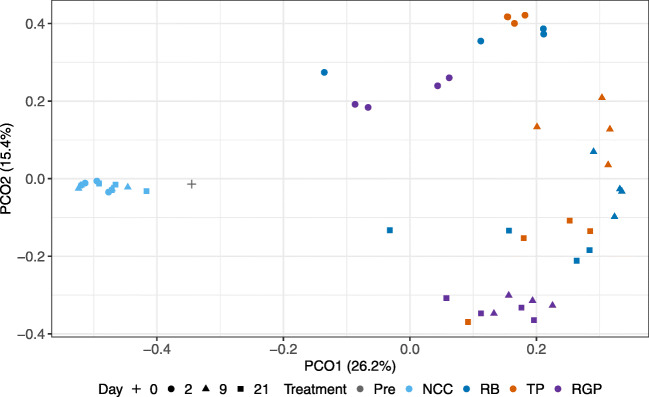
PCoA plot based on a Bray-Curtis dissimilarity matrix of ASV abundances

A repeated measures PERMANOVA of a Bray-Curtis distance matrix based on ASV abundances was conducted to test for significant effects of treatment and time on community structure. The results indicated there was an interaction between the factors (*F*_6,24_ = 3.30, *P* = 0.001) and that community composition changed significantly as a function of both treatment (*F*_3,24_ = 11.60, *P* = 0.001) and time (*F*_2,24_ = 9.88, *P* = 0.001). PCoA was used to visualize a Bray-Curtis distance matrix (Fig. 2). The first two dimensions of the PCoA captured 41.6% of variation in the dissimilarity matrix. The pre-treatment and NCC communities separated from ASD communities along the first axis. NCC communities did not separate by day, while ASD communities mostly clustered by day along the second axis. The lack of substantial change in NCC communities and drastic shifts in ASD communities over time is in line with observations in a recent ASD study with high temporal resolution (7 sampling points over 15 days) [8]. This study showed RB amendment induces turnover in the microbial community by linking changes in the microbiome and metabolome of ASD-treated soils. RB and TP communities tended to cluster together on all sampling days, similar to previous observations in field trials with these same carbon substrates [15]. RGP communities largely grouped separately from RB and TP communities. Also unlike RB and TP communities, the position of RGP samples in the ordination changed little between days 9 and 21. Together, PERMANOVA and PCoA indicate the significant effect of treatment was attributable to NCC and RGP communities differing from RB and TP. We also tested for homogeneity of group dispersions across time within treatment and found no support for early ASD communities exhibiting lower group variances. Only the dispersion of RB communities was significantly different between time points (*F*_2,9_ = 3.95, *P* = 0.033) due to the smaller variance of day 9 samples.

Our finding that group variances are largely unchanged over time highlights the importance of the identity and physiological potential of dormant microbes as determinants of the trajectory of microbial communities in ASD-treated soils [35]. It is likely the final composition of ASD communities in part reflects the initial presence of microbial taxa with the ability to rapidly respond to carbon input and adapt to reduced soil Eh, thereby establishing populations early on that continue to persist throughout the process [8]. Other major drivers of community composition would be soil physical and chemical characteristics [19, 34]. Because Eh reductions and changes in other physicochemical attributes (i.e., pH, accessible carbon pools, etc.) in ASD-treated soils are tightly coupled to microbial metabolisms [8, 31, 36], disentangling the contributions of the microbial “seed bank” and soil physicochemistry in determining the assemblage of ASD communities is difficult. Nonetheless, Liu et al. [19] identified soil physicochemistry and initial microbiota as the factors that are most important in shaping reassembled bacterial communities in ASD-treated soils.

ASD not only causes turnover in microbial communities but also reduces alpha diversity in many studies of the process [7, 12, 15, 34]. Alpha diversity is often negatively correlated with oxygen availability in marine ecosystems due to constraints on energy sources available to fuel microbial metabolism [37, 38]. In soil, the shift from aerobic to anaerobic conditions certainly reduces the niche space available to resident microbes. On the other hand, carbon addition may select for the proliferation of different groups based on their physiological capabilities and the chemical composition of substrates (i.e., carbon-to-nitrogen ratio, carbohydrate content, etc.) they are most adapted to use [39]. Therefore, we expected to observe reduced diversity in ASD soils relative to NCC and the initial communities. Additionally, we hypothesized that alpha diversity within the ASD treatments may increase over time as the community recovers from the initial disturbance and products of anaerobic metabolic reactions accumulate that may provide carbon and/or energy sources for microbes [40].

**Fig. 3:**
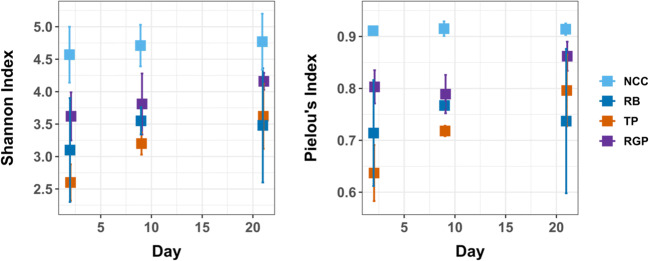
Alpha diversity measures: Shannon’s diversity index and Pielou’s evenness index

We found that Shannon diversity (5.01) and Pielou’s evenness (0.935) for the pre-treatment community was in the range of NCC communities (Fig. 3), indicating that diversity in the control mesocosms was largely unchanged relative to the initial community. Overall, Shannon diversity was significantly different between treatments (*χ*^2^_(3)_ = 28.25, *P* < 0.001) and changed over time (*χ*^2^_(2)_ = 11.66, *P* = 0.003), but there was no significant interaction effect (*χ*^2^_(6)_ = 5.94, *P* = 0.430). Treatment differences in Shannon diversity were mainly due to more taxa rich (*P* < 0.05) communities in NCC mesocosms in comparison to ASD mesocosms on all sampling days, although TP communities were significantly less diverse than RGP on day 2 (*P* = 0.021). Shannon diversity did increase in TP communities overtime (*P* = 0.007 for day 2 versus day 21 samples), but not in the other treatments. We obtained similar results for analysis of evenness (treatment, *χ*^2^_(3)_ = 34.88, *P* < 0.001; day, *χ*^2^_(2)_ = 8.21, *P* = 0.017) and there was a significant interaction between treatment and time (*χ*^2^_(6)_ = 13.07, *P* = 0.042). On the last sampling date, Pielou’s evenness was similar between NCC and RGP communities (*P* = 0.593). RGP communities also had higher evenness than TP on day 2 (*P* = 0.002) and RB on day 21 (*P* = 0.023).

Our results indicate that the reduction in alpha diversity in ASD communities is primarily due to carbon addition and not reduced soil Eh. We conclude this because NCC communities had soil Eh values that were indicative of anaerobic conditions and the cumulative anaerobicity of NCC soil was similar to ASD soils (Fig. 1). The strong shifts in the structure (e.g., beta diversity, Fig. 2) and reduced richness and evenness (Fig. 3) in soil microbial communities following ASD indicates there was a substantial turnover in the soil microbiome composition (as described below). The ability to detect reduced alpha diversity within a few days of the onset of ASD indicates that the conditions and physiological activities of microbes induced by the process work rapidly to kill microbial cells and cause degradation of DNA released upon cell death [8, 41, 42]. Microscopy techniques such as live/dead staining [43] and DNase activity assays [41] would be valuable for determining the viability of microbial populations and the stability of extracellular DNA in the earliest stages of the ASD. The significant increase in Shannon diversity for TP communities and trend of increasing richness and evenness in RGP communities shows that ASD carbon substrate amendment can result in more complex communities over time. It remains to be seen if diversity (compositional and functional) has consequences for pathogen suppression in ASD-treated soil.

### Taxonomic Composition of Soil Mesocosms

**Fig. 4:**
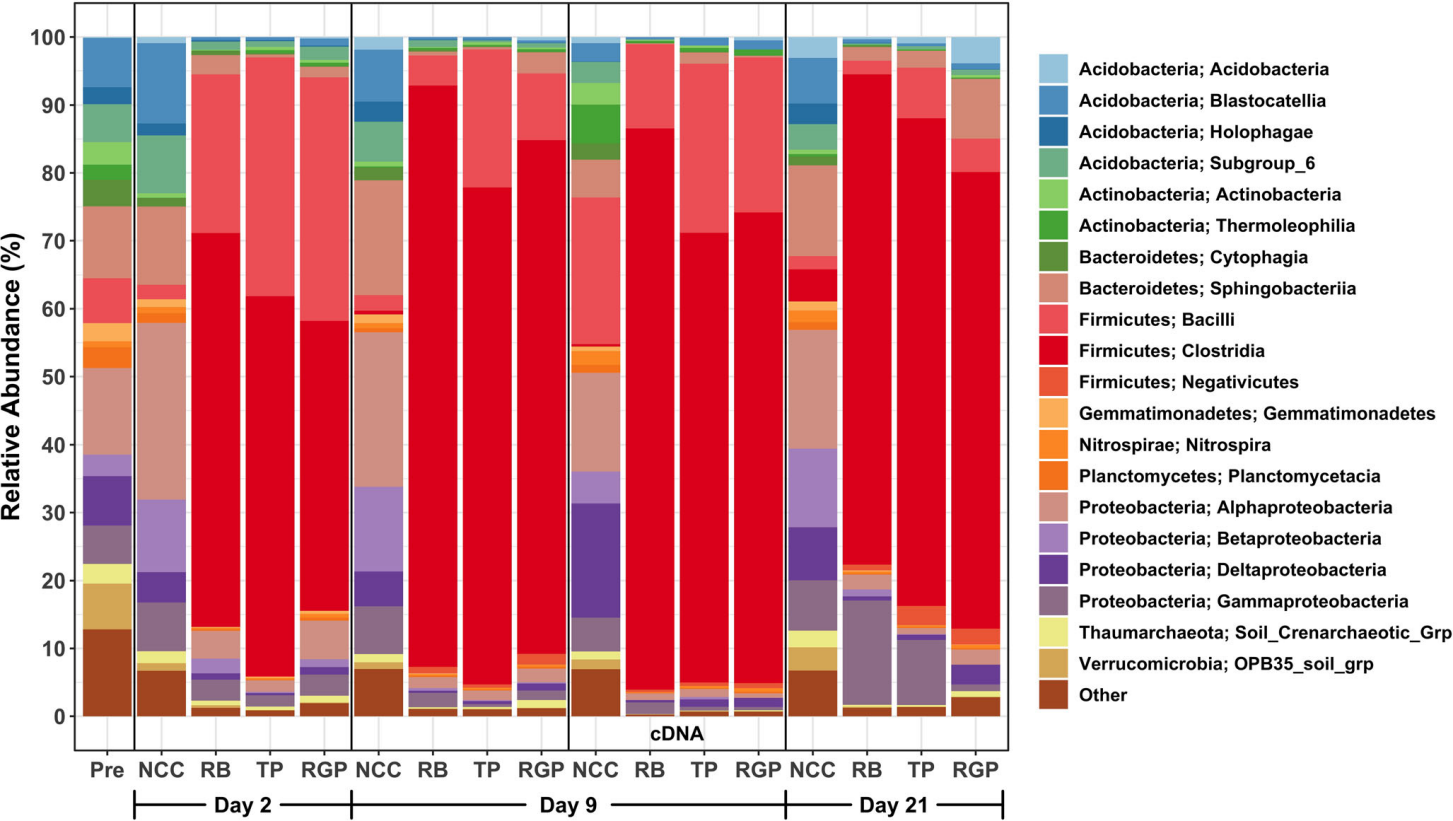
Relative proportions of the 20 most abundant classes in the soil microbiomes from the starting (pre) community, ASD mesocosms (RB, TP, RGP), and control mesocosms (NCC). On day 9, communities derived from amplicon sequencing of DNA and cDNA are shown

Pre-treatment, NCC, and ASD communities largely consisted of taxa belonging to Acidobacteria, Actinobacteria, Bacteroidetes, Firmicutes, and Proteobacteria, although the relative proportions of these groups differed between the soils (Fig. 4). The most notable difference between pre-treatment and NCC communities was the abundance of Alphaproteobacteria and Betaproteobacteria that were 10% and 7%, respectively more abundant in the day 2 NCC mesocosms than in pre-treatment soil. Over time, the relative abundances of most classes in NCC soils did not change drastically with the exception of members of the Acidobacteria that decreased from 23 to 16%. NCC communities also had consistently higher abundances of Bacteroidetes than ASD-treated soils. In ASD mesocosms, there was a large increase in the abundances of Firmicutes compared to pre-treatment and NCC soils. Within 2 days of ASD initiation, Firmicutes were at least 75% of the total community and maintained high relative proportions throughout the trial. This large increase in Firmicutes was due to the growth of bacteria belonging to Clostridia and Negativicutes, as members of the class Bacilli actually decreased in abundance over time in the ASD mesocosms. In our previous field study, we observed a similar, but less extensive (25 to 60%) increase in Firmicutes after 4 and 5 weeks of ASD treatment [15]. This points to the importance of identifying, isolating, and physiologically characterizing members of the Firmicutes that are key responders to conditions induced by ASD because these are the microbes that mediate pathogen suppression [8, 33, 44].

**Fig. 5:**
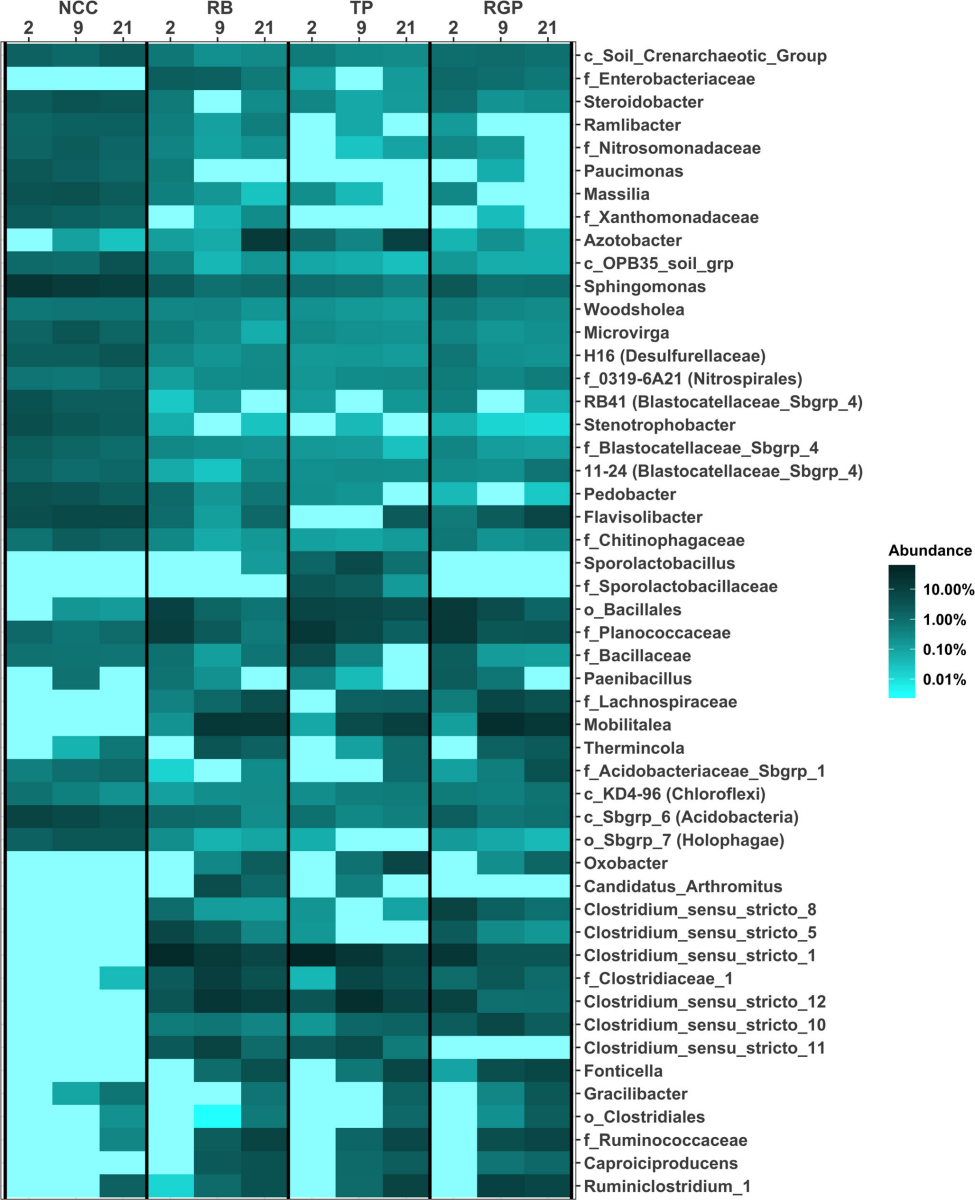
A heat map of the 50 most abundant genera in NCC and ASD mesocosms

To show how the soil microbiome changed over time in the NCC and ASD mesocosms at the taxonomic level, we graphed the relative proportions of the top 50 most abundant genera across all samples (Fig. 5). NCC communities largely consisted of aerobic microorganisms based on the taxonomic classification of genera; however, some of their abundances decreased over the 21-day incubation period (i.e., *Stenotrophobacter*, *Sphingomonas*, *Paucimonas*, *Massilia*, and *Pedobacter*). We also found that Desulfurellaceae H16 abundances increased over time in NCC mesocosms. This genus is thought to be a strict anaerobe but has been observed in aerobic lake sediments [45]. The heat map also shows that NCC mesocosms tended to lack genera belonging to the Clostridia and Negativicutes, although some of these taxa did appear in day 9 and 21 mesocosms (i.e., *Gracilibacter*). Given the gradual decline in soil Eh (Fig. 1), the abundance changes of these genera likely reflect reduced oxygen availability. The patterns in the change of genera abundances in ASD mesocosms were fairly similar between treatments for most members of the Firmicutes. RGP soils were different from RB and TP communities in the absence of several genera (i.e., *Clostridium* sensu stricto 11). In our previous field study, we identified 15 genera belonging to the Clostridia and Negativicutes as core responders (present in 90% of carbon-amended soils in both trials) to ASD implemented with different substrates, including RB and TP [15]. We lacked data on how the abundances of these core responders changed during ASD, as the genera were identified based on their presence in post-treatment soils. By sampling over time in this study, we are able to detect that some of the core responders we identified in our field trial exhibited decreases in abundance over the course of treatment (e.g., *Clostridium* sensu stricto 1, 10, and 12; Fig. 5). We also observed an increase in abundances of 7 core genera over the course of ASD using RB, TP, and RGP in this greenhouse study (e.g., *Caproiciproducens*, *Fonticella*, *Gracilibacter*, *Mobilitalea*, *Ruminiclostridium_1*, unclassified Ruminococcaceae, and *Thermincola*). *Caproiciproducens*, *Fonticella*, and *Mobilitalea* were found only in ASD treatments in this trial and isolates from these genera are all capable of producing acetate [46–48], which is known to inhibit plant pathogens when added to soil at concentrations similar to those in ASD-treated soils [10].

**Fig. 6:**
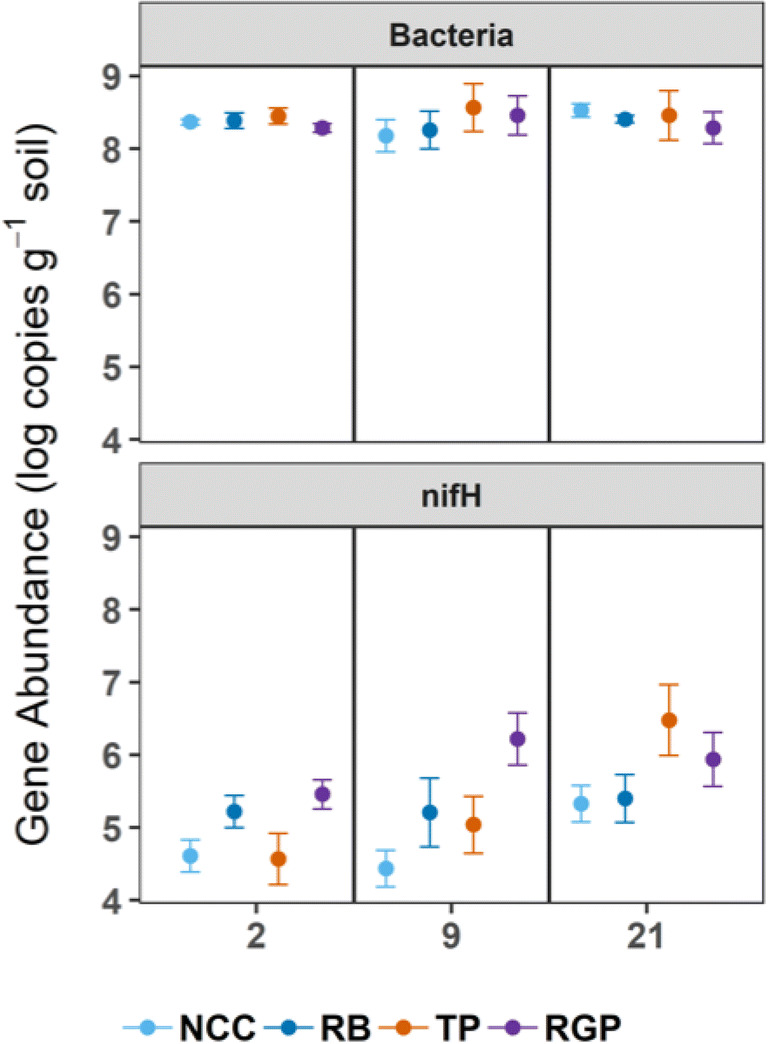
Bacterial 16S rRNA gene and nitrogenase (*nifH*) gene abundances in NCC and ASD soil microbiomes

In our previous trial, we detected an increase in the genomic potential for biological nitrogen fixation based on a predicted metagenomic analysis and high abundances of genera such as *Azotobacter* and *Azospira* in post-ASD soils [15]. Similar to the response of Firmicutes, we again found that *Azotobacter* abundances increased from < 1 to ~ 10% in RB and TP mesocosms. Quantitative PCR confirmed that *nifH* abundances increased over time in ASD-treated soils (Fig. 6), especially in TP and RGP mesocosms. We then calculated correlations between genera and *nifH* abundances to determine if there were any significant relationships. In addition to *Azotobacter*, we identified *Oxobacter*, *Desulfotomaculum*, and *Desulfosporosinus* abundances as being significantly correlated with *nifH* abundances. These genera also have the genomic potential for nitrogen fixation [49–51]. Our results here and in the field trial indicate that nitrogen fixation may be an operational biogeochemical process in ASD-treated soils and requires experimental validation in both greenhouse and field settings.

Finally, on day 9, we compared soil microbiomes derived from DNA and RNA (cDNA) extractions to determine if there was a difference in the present versus active components of the communities (Fig. 5). NCC communities exhibited the largest disparity in the relative proportions of the major classes of bacteria. For example, the class Bacilli was 2.3% of the DNA-based community and 21.6% of the cDNA-based libraries. This pattern was also observed for Deltaproteobacteria in NCC soils (5.1% in DNA libraries and 16.8% of cDNA libraries). Conversely, Sphingobacteriia had a lower abundance in cDNA libraries (5.5%) in comparison to DNA libraries (16.9%). Several genera belonging to these classes were detected as differentially abundant (i.e., an unclassified group belonging to the order Bacillales and a deltaproteobacterium, *Sorangium*). For the most part, ASD-treated soils did not exhibit significant differences in the abundances of genera between the cDNA and DNA libraries. At the class level, there were disparities in the relative abundances of Bacilli in RB mesocosms, Clostridia in TP soils, and both groups in RGP samples. In these cases, Bacilli were more abundant in cDNA libraries and Clostridia were less abundant in cDNA samples. These results suggest that RNA-based approaches should provide a more nuanced picture of the potential activity of soil microbiome members in ASD-treated soils, especially when the difference between control and treated soils is due only to carbon addition.

## Conclusion

ASD results in drastic shifts in the soil microbiome that can be dependent on the carbon source used to initiate the process. In this study, we designed soil mesocosms that could be repeatedly sampled and maintain soil Eh levels similar to those in ASD field trials to track shifts in the structure and composition of soil microbial communities over time. This enabled us to determine that taxa which we had identified as core responders to ASD carbon amendment under field conditions do indeed increase in abundance over time under controlled growth chamber conditions. These taxa are targets for enrichment and isolation to investigate and confirm their role in pathogen suppression. Moreover, we used qPCR to confirm that taxa with the genomic potential for nitrogen fixation are also highly responsive to ASD treatment. This raises the possibility for interactions between nitrogen metabolism and fermentative metabolisms that are thought to be important in pathogen control mechanisms. The implications of the growth over time of nitrogen-fixing microbes in ASD-treated soils for plant nutrition also remain to be studied, but potentially offers a mechanism for increasing nitrogen availability.
